# 

**Published:** 1869-02-15

**Authors:** 


### *CONTENTS.*

TRANSLATIONS:

Memoir upon the Peripheral Terminations of Motor Nerves in the Animal Series. Translated expressly for THE JOURNAL, by W. Hay, M. D. Associate Editor........ 97

Pathogenesis. Contagion of Phthisis Pulmonalis........ 98

CORRESPONDENCE:

Clinical Surgery in the Paris Hospitals........ 102

Our California Letter........ 105

ORIGINAL COMMUNICATIONS:

Puerperal Metro Peritonitis. By J. W. Dora, M. D., Mattoon, Ills........ 110

Super-Oxygenation as a Therapeutic Measure, with Notes of Cases Treated. By S. S. Wallihan, M. D., Chicago........ 116

Sun Stroke. By M. Waterhouse, M. D., Portage City, Wis........ 121

FOREIGN ITEMS:

A New Method of Laryngoscopy—Animal Grafts........ 123

PROCEEDINGS OF SOCIETIES:

Proceedings of the Chicago Medical Society........ 125

BOOKS RECEIVED:

Opthalmic Surgery and Treatment, with Advice on the Use and Abuse of Spectacles. By John Phillips, Optician and Occulist........ 128

